# A panel of glycoproteins as candidate biomarkers for early diagnosis and treatment evaluation of B-cell acute lymphoblastic leukemia

**DOI:** 10.1186/s40364-016-0055-6

**Published:** 2016-01-27

**Authors:** Marcio de Souza Cavalcante, José Camilo Torres-Romero, Marina Duarte Pinto Lobo, Frederico Bruno Mendes Batista Moreno, Leonardo Primo Bezerra, Diego Silva Lima, Jesamar Correia Matos, Renato de Azevedo Moreira, Ana Cristina de Oliveira Monteiro-Moreira

**Affiliations:** Northeast Network of Biotechnology (RENORBIO), State University of Ceará, Fortaleza, Ceará Brazil; Center of Experimental Biology (NUBEX), University of Fortaleza (UNIFOR), Fortaleza, Ceará Brazil; Department of Biochemistry and Molecular Biology, Federal University of Ceará, Fortaleza, Ceará Brazil; Development and Technological Innovation in Drug Program, Federal University of Ceará, Fortaleza, Ceará Brazil; Reference Center at Children’s Cancer Diagnosis and Adolescents Dr. Murilo Martins, Albert Sabin Hospital, Fortaleza, Ceará Brazil

**Keywords:** Acute lymphoblastic leukemia, Biomarker, Lectin, Frutalin, Mass spectrometry

## Abstract

**Background:**

Acute lymphoblastic leukemia is the most common malignant cancer in childhood. The signs and symptoms of childhood cancer are difficult to recognize, as it is not the first diagnosis to be considered for nonspecific complaints, leading to potential uncertainty in diagnosis. The aim of this study was to perform proteomic analysis of serum from pediatric patients with B-cell acute lymphoblastic leukemia (B-ALL) to identify candidate biomarker proteins, for use in early diagnosis and evaluation of treatment.

**Methods:**

Serum samples were obtained from ten patients at the time of diagnosis (B-ALL group) and after induction therapy (AIT group). Sera from healthy children were used as controls (Control group). The samples were subjected to immunodepletion, affinity chromatography with α-d-galactose-binding lectin (from *Artocarpus incisa* seeds) immobilized on a Sepharose^TM^ 4B gel, concentration, and digestion for subsequent analysis with nano-UPLC *tandem* nano-ESI-MS^E^. The program *Expression*^E^ was used to quantify differences in protein expression between groups.

**Results:**

A total of 96 proteins were identified. Leucine-rich alpha-2-glycoprotein 1 (LRG1), Clusterin (CLU), thrombin (F2), heparin cofactor II (SERPIND1), alpha-2-macroglobulin (A2M), alpha-2-antiplasmin (SERPINF2), Alpha-1 antitrypsin (SERPINA1), Complement factor B (CFB) and Complement C3 (C3) were identified as candidate biomarkers for early diagnosis of B-ALL, as they were upregulated in the B-ALL group relative to the control and AIT groups. Expression levels of the candidate biomarkers did not differ significantly between the AIT and control groups, providing further evidence that the candidate biomarkers are present only in the disease state, as all patients achieved complete remission after treatment.

**Conclusion:**

A panel of protein biomarker candidates has been developed for pre-diagnosis of B-ALL and also provided information that would indicate a favorable response to treatment after induction therapy.

## Background

Acute lymphoblastic leukemia (ALL) is the most common malignant cancer in childhood, and is responsible for approximately 25 % of all childhood cancers and 72 % of all cases of pediatric leukemia [1]. The current standards for diagnosis of ALL integrate the study of cell morphology, immunophenotyping, and genetics/cytogenetics, as described in the classification of lymphoid cancers published by the World Health Organization (WHO) in 2008 [2]. Of lymphoid cancers, as designated using the most recent WHO classification, the purely leukemic presentation, B-lineage ALL (85 %) is the most common [3]; it will be addressed in this study. The signs and symptoms of childhood cancer are very challenging to identify, as it is not the first diagnosis to be considered for nonspecific complaints, leading to potential uncertainty in diagnosis. Moreover, children showing the first signs of cancer frequently do not appear severely ill, which may delay diagnosis. In addition, childhood cancer can mimic other common childhood diseases and even normal developmental physiological processes [4]. Measures to improve cancer incidence rates in adults include prevention of exposure to known carcinogenic risk factors, such as smoking, but environmental factors play a very small role in the development of childhood cancers. Thus, primary prevention measures are not effective in averting the development of cancer in this age group, and secondary prevention, i.e. early diagnosis, is therefore essential [5]. In the specific case of ALL, early diagnosis and treatment increase the chances of a cure [4]. Affinity chromatography with α-D-galactose-binding lectin from *Artocarpus incisa* immobilized on a Sepharose™ 4B gel, combined with identification and quantification of glycoproteins by mass spectrometry, are excellent tools for comparative serum studies. The biomarker pipeline is commonly viewed as a series of preclinical phases: biomarker discovery, and verification before the final clinical evaluation. The comparative analysis results in a list of hundreds of proteins that are differentially-expressed between healthy and diseased samples [6]. In this study, the preclinical phase of biomarker discovery was applied and a proteomic analysis of serum samples from pediatric patients with B-ALL was performed, to analyze levels of glycoprotein expression, with the aim of identifying biomarkers to aid in the early diagnosis of B-ALL and to assess the response to induction therapy.

## Methods

### Patients and samples

Serum samples were collected from ten pediatric patients with B-ALL at diagnosis and after induction therapy. These patients were diagnosed based on morphological, immunophenotypic, and genetic tests. The study population was composed mainly of children from the lower middle class, who attended a reference hospital for the diagnosis and treatment of childhood cancers in the State of Ceará -Brazil. The mean age of the patients was 6.15 years (*n* = 10); the average age of female patients was 3.8 years (*n* = 6) and that of male patients was 3.5 years (*n* = 4). All patients were assigned as low risk (LR) when aged from 1 to 9 years old, with a white blood cell (WBC) count <50 × 10^9^/L, and no CNS involvement. The risk stratification and chemotherapy were performed according to the protocol of the Brazilian Group for Treatment of Childhood Leukemia (GBTLI-LLA-2009). All patients experienced complete clinical and morphological remission and are presently surviving. The serum samples used as controls were obtained from ten non-leukemic pediatric patients. All samples were stored at −80 °C until use and concentrations were determined using a Nanovue Plus™ apparatus (GE Healthcare, Uppsala, Sweden). The study was conducted with the approval of the Research Ethics Committee at the Hospital Infantil Albert Sabin, associated with the Secretary of Health of the State of Ceará.

### Serum preparation

For each sample, high-abundance human serum albumin (HSA) and immunoglobulin G (IgG) proteins were depleted using a HiTrap™ Albumin & IgG Depletion column (GE Healthcare) on an ÄKTA purifier 10 Fast protein liquid chromatography (FPLC) system (GE Healthcare) according to the manufacturer’s instructions. After removal of high-abundance proteins, the flow-through fractions were collected and stored at −80 °C until use.

### Lectin affinity chromatography

The serum-free high-abundance proteins were thawed, centrifuged for 15 min at 12,000 × *g* at 8 °C, filtered through a 0.22-μM membrane (Vertipure™ PVDF syringe filters, Veritical) and applied to a 5-mL column packed with Sepharose-Frutalin, prepared as mentioned previously, in a XK16 column on an ÄKTA purifier 10 FPLC system (GE Healthcare). The column was washed with five CV of buffer A (20 mM Tris–HCl, pH 7.4), and the lectin-bound proteins were eluted with four CV of elution buffer B (20 mM Tris–HCl, pH 7.4, with 0.2 M galactose). The eluted protein solution was dialyzed and concentrated by spinning at 8000 × *g* (Vivaspin® 6, with a molecular weight cut-off of 3 kDa, GE Healthcare), and used for further analyses.

### Proteomic analysis

Briefly, each sample containing 50 μg of protein was denatured with 0.2 % RapiGest™ SF (Waters, Milford, USA), reduced with 10 mM dithiothreitol, alkylated with 10 mM iodoacetamide, and enzymatically digested with trypsin (Promega, Madison, WI, USA). At the end of this process, the samples were centrifuged and the supernatant was transferred to new vials, to which 5 μL of internal standard, alcohol dehydrogenase (ADH, 50 fmol, access code P00330 in SwissProt) and 85 μL of 3 % acetonitrile solution with formic acid 0.1 % were added. The final glycoprotein and ADH concentrations were estimated to be 250 ng/μL and 25 fmol/μL, respectively, in a final volume of 200 μL. The quantitative and qualitative nano-UPLC *tandem* nano-ESI-MSE experiments were performed on digested samples using peptide reversed-phase chromatography with 3 to 40 % (*v/v*) of acetonitrile containing 0.1 % formic acid for 90 min, maintained at a flow rate of 600 nL/min for 100 min in a nanoACQUITY UPLC core system. A nanoACQUITY C18 UPLC BEH 1.7 μm, 100 μm × 10 cm reversed-phase column was used in conjunction with an SCX 5 μm, 180 μm × 23 mm pre-column. All analyses were performed with electrospray ionization in positive ion ESI(+) mode and a NanoLockSpray source. Data-independent acquisition (MS^E^) was performed with a SYNAPT HDMS G1 mass spectrometer (Waters, Manchester, UK), programmed to automatically switch between standard MS (3 eV) and high-energy collision MS^E^ (12–50 eV), applied to trap ‘T-wave’ CID (collision-induced dissociation) cells, in the presence of argon gas. The transfer from the collision cell was adjusted to 1 eV, with 1 s, at both low and high energy. After the time of flight analysis (TOF), m/z 50 to 3000 spectra were collected. However, the RF offset of the quadrupole was set for efficient acquisition of LC/MS data from m/z 300–3000, ensuring that any mass less than m/z 300 observed in the LC/MSE data stemmed only from the collision cell. Thus, the low-mass values were known CID fragmentation products, not the result of source fragmentation. For processing of spectra and database searches, the Protein Lynx Global Server (PLGs) package v. 2.4, containing the Expression^E^ v. 2.4 program, was used. The PLGs used a new algorithm to process raw data obtained by MS using ion properties, i.e. retention time, intensity of precursor/productions, and exact mass. A label-free proteomic approach was used for the quantitative analysis. Then the PLGs generated a list of all precursors and products. This list contained the mass of the precursor and product ions for each peptide, to be searched against the non-redundant proteins database, UniProtKB/Swiss-Prot 57.1, under search conditions based on taxonomy [Human (*Homo sapiens*)]. For each protein, the program Expression^E^ selected all corresponding peptides from the samples and compared the intensities of these for relative protein quantification. Using the intensity of a peptide of known quantity, ADH, the program performed self-standardization of data sets. Lists of proteins were then filtered to show only those present in all three repeated injections of each sample, from which an output table was created. This table showed the names, access codes, and expression levels of the proteins, and indicated whether they were up-regulated ≥2-fold, down-regulated ≤0.5-fold, or whether they did not show significant differences between the groups (unchanged), 0.5< expression level <2.

### Protein interaction network analysis

The differentially expressed proteins were used for pathway analysis. Swiss-Prot accession numbers were inserted into the Search Tool for the Retrieval of Interacting Genes/Proteins (STRING) software, version 9.05, which is available at http://string.embl.de/, with the following analysis parameters: *Homo sapiens*, confidence level 0.400–0.900, using the active prediction method [7].

## Results and discussion

### Patient characteristics

The demographic and clinical data for the patients are summarized in Table 1. To form the panel of candidate proteins for early biomarkers and demonstrate their expression profiles, the pediatric patients were evaluated at two different times: at diagnosis (B-ALL Group; *n* = 10) and after induction therapy (AIT Group; *n* = 10). Samples of healthy children (Control Group; *n* = 10) were obtained for comparison.

**Table 1:** Summary of characteristics of patients with B-ALL

Code Assigned	Gender	Age at Diagnosis	FAB Classification	Immunophenotypical Classification	Karyotype	Risk Group	MRD	Treatment Outcome
P1	M	3	L1	Common	Absence of metaphases	LR	_	CR
P2	F	3	L1	Pre-B	Absence of metaphases	LR	_	CR
P3	F	5	L1	Common	56,XX,+X,+4,+6,+8,+10,+11,+14,+17,+21,+mar/46,XX	LR	_	CR
P4	M	2	L1	Common	46,XY	LR	_	CR
P5	F	2	L1	Common	46,XX	LR	_	CR
P6	F	3	L1	Common	54,XX,+X,+6,+15,+15,+17,+18,+21,+21/46,XX	LR	_	CR
P7	F	5	L1	Common	47,XY,+21 c	LR	_	CR
P8	M	6	L1	Common	Absence of metaphases	LR	_	CR
P9	F	5	L1	Pre-B	46, XX	LR	_	CR
P10	M	3	L1	Pre-B	46,XY	LR	_	CR

Code: Internal register assigned for the study; *M* Male, *F* Female, *LR* Low Risk, *MRD* Minimum Residual Disease, *CR* Complete remission

### Reduction of dynamic range

**Fig. 1:**
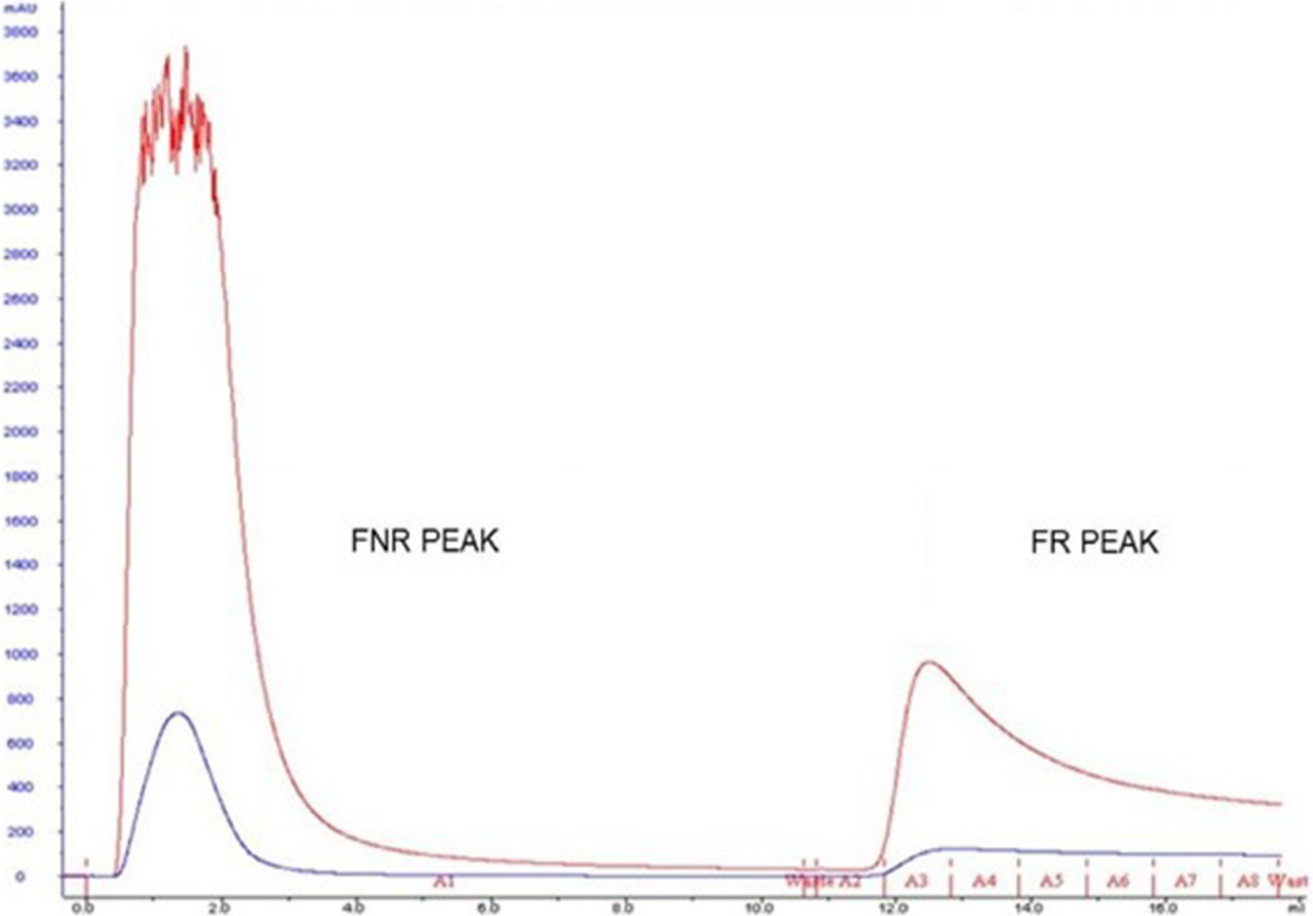
Graphical representation of the affinity chromatography process on a Frutalin-immobilized column with Sepharose 4B, coupled with an ÄKTA purifier 10 FPLC system. Peak I represents the non-retained fraction (FNR) and Peak II represents the retained fraction (FR). The fractions were obtained after elution with their respective buffers: 20 mM Tris–HCl, pH 7.4, in0.15 M NaCl (Buffer A) and 0.2 M galactose and 20 mM Tris–HCl, pH 7.4, in 0.15 M NaCl (Buffer B). The blue line represents absorbance at 280 nm and the red represents emission at 216 nm

The depletion of high-abundance proteins in serum, HSA and IgG, followed by affinity chromatography with the plant lectin Frutalin immobilized on Sepharose™ 4B (Fig. 1), reduced the dynamic range and increased the capacity to identify lower-abundance proteins. The retained fraction (FR) peak containing the protein of interest was concentrated and digested, for later analysis by nano-LC-MS/MS.

### Proteomic analysis

**Fig. 2:**
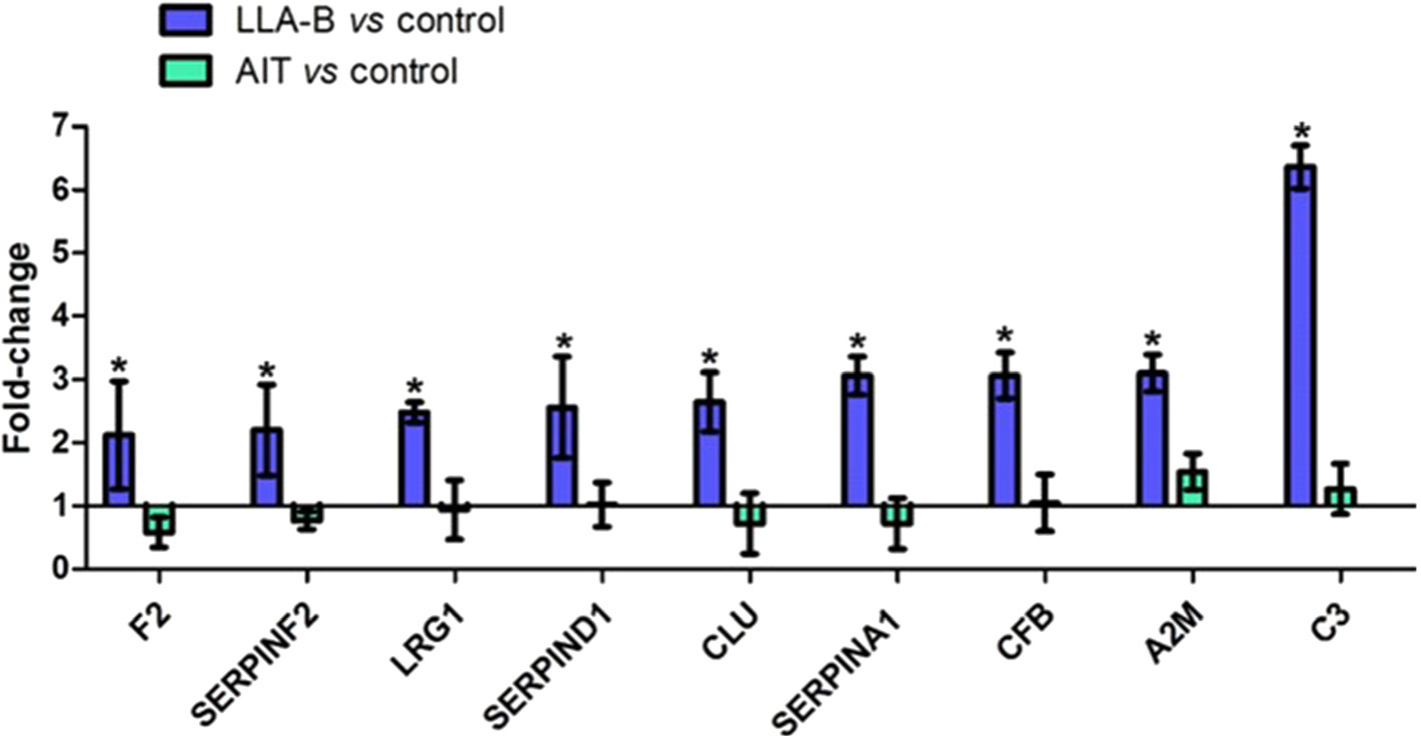
Panel of candidate protein biomarkers for B-ALL. Blue columns represent the expression levels of the proteins in B-ALL patients at the time of diagnosis in relation to the control. Green columns represent the expression levels of the proteins in B-ALL patients after induction therapy (day 35) relative to controls. (*) (*p* < 0.05)

In the proteomic analysis, a total of 96 proteins were identified. Leucine-rich alpha-2-glycoprotein 1 (LRG1), Clusterin (CLU), thrombin (F2), heparin cofactor II (SERPIND1), alpha-2-macroglobulin (A2M), alpha-2-antiplasmin (SERPINF2), Alpha-1 antitrypsin (SERPINA1), Complement factor B (CFB) and Complement C3 (C3) were over-expressed in the B-ALL compared to the control and AIT groups, and were therefore identified as candidate biomarkers for early diagnosis of B-ALL. The AIT group showed no significant differences in the expression levels of these proteins, compared to the control group, did not show any significant change in the level of expression of these proteins, a fact that further reaffirms the presence of these potential biomarkers in a disease state, as all patients achieved complete remission after treatment (Fig. 2).

LRG1 was 2.16-fold upregulated in the B-ALL group compared to the Control group. LRG1 is involved in cell adhesion and development, protein-protein interaction, and signal transduction. Studies have shown that it may be a biomarker for ovarian cancer, pancreatic cancer, oral squamous cell carcinoma [8, 9], and non-small cell lung cancer [10]. CLU, also called apolipoprotein J, was 2.64-fold upregulated in the B-ALL group compared to the control. CLU is expressed in a variety of tissues, with a ubiquitous pattern of expression. It has a high sensitivity to external stress stimuli. It has been reported to function in complement inhibition, clearance of cellular debris, folding of denatured proteins (chaperone), and regulation of cell death [11, 12]. Several studies have shown that it is over-expressed in many serious physiological conditions, including degenerative kidney disease and various neurodegenerative conditions. CLU also plays an important role in tumorigenesis and progression of many human cancers [13]. Higher levels of this protein have been observed in the progression of prostate cancer and renal carcinoma compared to normal tissues [14, 15] and it was shown to be over-expressed in anaplastic large cell lymphomas [16], ovarian cancer [17], esophageal cancer [18], and colorectal cancer; it has also been implicated in pre-diagnosis of colorectal cancer [19]. The accumulation of CLU protein is correlated with the aggressiveness of certain breast tumors [20].

Our results also confirm the important relationship between cancer and phenomena associated with blood coagulation. Several studies have reported that approximately 50 % of patients with malignant disease and more than 90 % of those that evolve to metastasis present evidence of abnormalities in coagulation and/or fibrinolysis [21–25]. Several of the proteins identified in this study, such as F2, SERPINF2, SERPIND1, A2M, are known to be involved in coagulation; here, they were found to be overexpressed by 2.12-, 2.20-, 2.56- and 3.10-fold. Cancerous cells can activate the coagulation system directly, by generating thrombin, or indirectly, by stimulating mononuclear cells to express a variety of pro-coagulant proteins [25]. These pro-coagulant factors are expressed in cancerous cells, which results in the activation of coagulation factors VII and X [26, 27]. Cytokines released from cancer cells trigger coagulant activity in monocytes, platelets, and endothelial cells. The formation of fibrin can occur in many types of cancer tissues, and fibrin matrix formation seems to favor protection of cancer cells from the immune system [28]. Although this study reports over-expression of coagulation-associated proteins in patients with B-ALL, which is a malignant tumor of circulating cells, fibrinogen may not be involved in immune protection of non-adherent cancers, but may function as a promoter of the growth and spread of cancer [26, 29, 30]. Some studies have reported an association between hemostatic abnormalities and gastric cancer [31, 32], and an association of cancer with thrombotic events [33–38]. Other studies have shown that blood levels of fibrinogen are correlated with aggressiveness in gastric cancer [21, 35]. Kwon et al. [21] reported that a single determination of coagulation markers, in particular thrombin-antithrombin complex (TAT), fibrin monomer, and D-dimer, is sufficient to determine the prognosis and survival of patients with cancer. Over-expression of anticoagulant proteins may be due to stimulation by the excess of thrombin in blood [21]. Braoudaki et al. [39] reported differentially increased expression levels of A2M in bone marrow plasma samples in patients with ALL. In our study this protein was 3.1-fold upregulated in the serum of B-ALL patients, and its level of expression decreased significantly after induction therapy. Nevertheless, compared with serum samples from control patients, no significant changes in expression levels of A2M were observed. This shows that A2M is in fact upregulated in patients with B-ALL and can be considered both as a candidate biomarker of disease as well as for evaluation of the response to induction therapy. Moreover, serum analysis is less invasive than bone marrow biopsy [39].

Alpha-1 antitrypsin (SERPINA1) was 3.06-fold upregulated compared to controls. SERPINA1 is a serum glycoprotein that is synthesized primarily in the human liver and in macrophages. It belongs to the serpin family, which plays a central role in the control of degradation of tissues through its inhibitory effect on neutrophil elastase and other serine proteases, including trypsin, chymotrypsin, cathepsin G, plasmin, thrombin, tissue kallikrein, and activated factor X (FXa) [40–42]. Many types of cancer cells have been shown to express and secrete alpha-1 antitrypsin [43–45]. In addition, high serum levels of this protein have been reported in a number of inflammatory diseases and in various malignancies, such as hepatocellular carcinoma, multiple myeloma, pancreatic carcinoma, prostate carcinoma, primary carcinoma of the lung, cervical carcinoma, gastric cancer, laryngeal cancer, nasopharyngeal carcinoma, breast cancer, and colorectal cancer [45–52]. Some studies reported that levels of this protein were correlated with cancer stage [47, 53–56]. A previous study of lung and prostate cancers, showed a direct correlation between high serum levels of SERPINA1 and cancer stage. It has been also suggested that the main source of the increase in blood levels of this protein in cancer patients is the growth of cancer cells [47]. In a recent survey, also of lung cancer and prostate cancer, a decrease in the serum levels of SERPINA1 was observed several weeks after treatment, suggesting that it may be an important indicator of the effectiveness of cancer treatment [57].

Complement factor B (CFB) and Complement C3 (C3), which are both associated with the complement system, were found to be 3.06- and 6.36-fold upregulated. The complement system is an essential part of the innate immune response that participates in the elimination of altered cells from the body. Due to the large number of genetic and epigenetic alterations associated with carcinogenesis, neoplastic transformation can increase the ability of malignant cells to activate this system [58], which may explain the over-expression of these proteins, in agreement with other studies that report the over-expression of complement proteins in pulmonary cells [59, 60]. Although malignant cells often develop mechanisms for protection against and resistance to complement, recent research on the mechanisms regulating complement activity demonstrated that the over-expressed proteins showed inhibitory activity [61–63]. Therefore, the proteins found to be upregulated in this study may be candidate biomarkers of B-ALL, because the alteration in expression was observed only in cancer patients. This result is corroborated by the absence of changes in expression levels of these proteins in the serum of patients who underwent induction therapy and who experienced complete remission, as well as in healthy individuals from the control group.

**Fig. 3:**
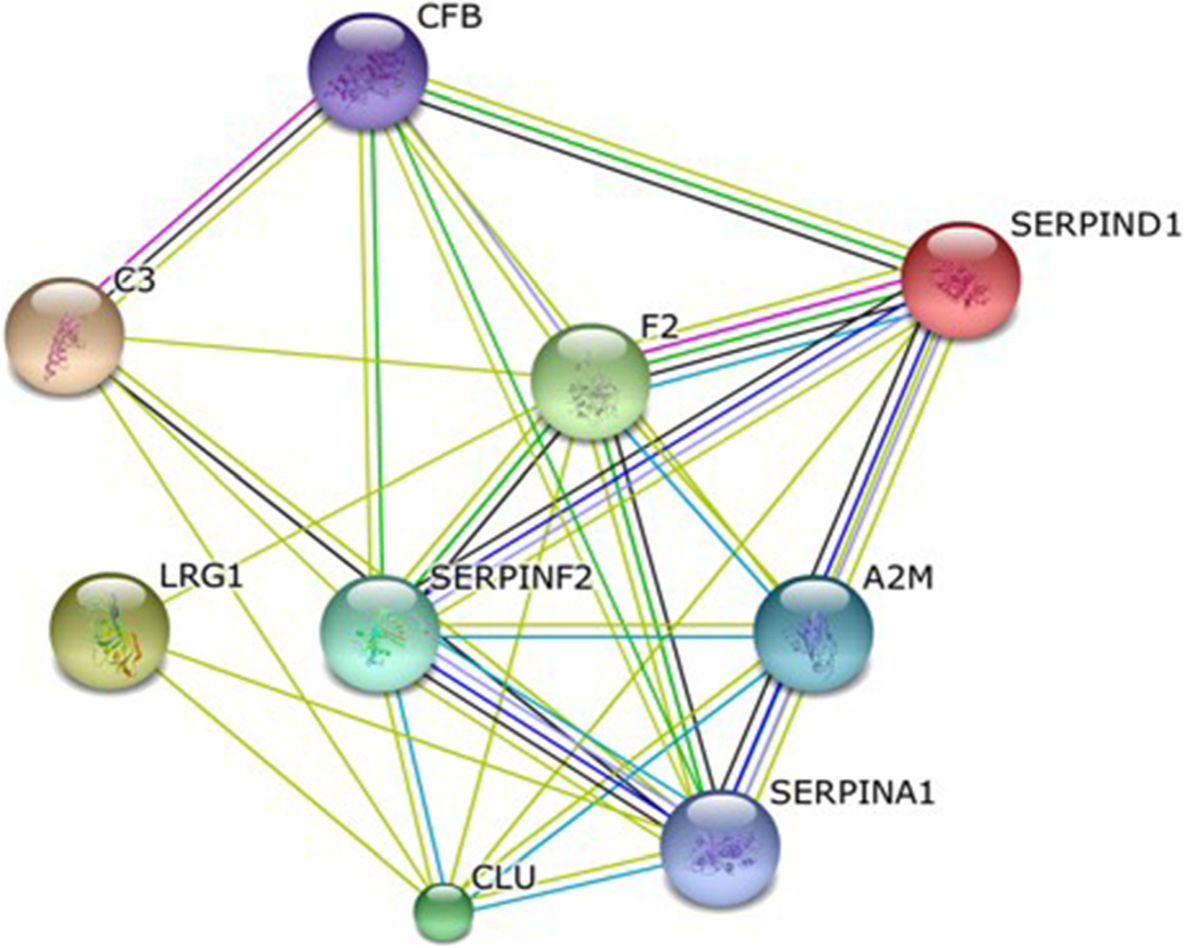
Interactome generated by STRING interaction database. Required confidence (score): medium confidence (0.400)

All nine proteins identified found to be upregulated in the B-ALL group showed evidence of interaction, as shown by the protein interaction network generated by the STRING interaction database (http://string-db.org/) (Fig. 3). Some studies have shown that increased expression of thrombin is associated with leukemia, as well as several other diseases [64, 65]. Similarly, as previously mentioned, the increase in blood levels of SERPINA1 in cancer patients is proportional to the growth of cancer cells; a decrease is observed several weeks after chemotherapy. Because these proteins lack specificity for the diagnosis of B-ALL if used in isolation, we suggest the creation of panel of biomarker.

## Conclusion

LRG1, CLU, F2, SERPIND1, A2M, SERPINF2, SERPINA1, CFB, and C3 were identified as candidate biomarkers for early diagnosis of B-ALL; all were over-expressed in the B-ALL group compared to the control and AIT groups. The AIT group did not display any significant changes in the expression levels of these proteins, compared to the control group. All patients in the AIT group achieved complete remission after treatment; this indicates that these biomarkers are only present in the disease state. These candidate biomarkers may improve the pre-diagnosis of B-ALL, which is currently difficult to diagnose in the early stages; the biomarkers may also provide key information on the response to treatment after induction therapy. Further clinical studies are necessary to determine whether over-expression of these proteins is associated with development of acute symptoms in patients with suspected ALL. These markers may help to determine response to treatment and to improve the survival of children with this disease.

## Abbreviations

ALL: Acute lymphoblastic leukemia
WHO: World Health Organization
LR: Low Risk
WBC: White Blood Cell
HSA: Human Serum Albumin
IgG: Immunoglobulin G
FPLC: Fast protein liquid chromatography
ADH: alcohol dehydrogenase
STRING: Search Tool for the Retrieval of Interacting Genes/Proteins
LRG1: Leucine*-*rich alpha*-*2*-*glycoprotein 1
CLU: Clusterin
F2: thrombin
SERPIND1: heparin cofactor II
A2M: alpha-2-macroglobulin
SERPINF2: alpha-2-antiplasmin
SERPINA1: Alpha-1 antitrypsin
CFB: Complement factor B
C3: Complement C3
TAT: Thrombin-antithrombin complex
